# Effect of Working from Home on the Association between Job Demands and Psychological Distress

**DOI:** 10.3390/ijerph19106287

**Published:** 2022-05-22

**Authors:** Hisashi Eguchi, Akiomi Inoue, Ayako Hino, Mayumi Tsuji, Seiichiro Tateishi, Kazunori Ikegami, Tomohisa Nagata, Ryutaro Matsugaki, Yoshihisa Fujino, on behalf of the CORoNaWork Project

**Affiliations:** 1 Department of Mental Health, Institute of Industrial Ecological Sciences, University of Occupational and Environmental Health, Japan, Kitakyushu 807-8555, Japan; ayako-hino@med.uoeh-u.ac.jp; 2 Institutional Research Center, University of Occupational and Environmental Health, Japan, Kitakyushu 807-8555, Japan; akiomi@med.uoeh-u.ac.jp; 3 Department of Environmental Health, School of Medicine, University of Occupational and Environmental Health, Japan, Kitakyushu 807-8555, Japan; tsuji@med.uoeh-u.ac.jp; 4 Disaster Occupational Health Center, Institute of Industrial Ecological Sciences, University of Occupational and Environmental Health, Japan, Kitakyushu 807-8555, Japan; tateishi@med.uoeh-u.ac.jp; 5 Department of Work Systems and Health, Institute of Industrial Ecological Sciences, University of Occupational and Environmental Health, Japan, Kitakyushu 807-8555, Japan; kikegami@med.uoeh-u.ac.jp; 6 Department of Occupational Health Practice and Management, Institute of Industrial Ecological Sciences, University of Occupational and Environmental Health, Japan, Kitakyushu 807-8555, Japan; tomohisa@med.uoeh-u.ac.jp; 7 Department of Preventive Medicine and Community Health, School of Medicine, University of Occupational and Environmental Health, Japan, Kitakyushu 807-8555, Japan; pt-matsugaki@med.uoeh-u.ac.jp; 8 Department of Environmental Epidemiology, Institute of Industrial Ecological Sciences, University of Occupational and Environmental Health, Japan, Kitakyushu 807-8555, Japan; zenq@med.uoeh-u.ac.jp

**Keywords:** COVID-19 pandemic, psychological distress, psychosocial factors, work from home, job demand–resource model

## Abstract

Limited information is available regarding the association between workplace psychosocial factors and general mental health status among workers during the coronavirus disease 2019 pandemic. This study examined how working from home affected the association between job demands and psychological distress (PD). A cross-sectional online survey was conducted in December 2020 (N = 27,036). The dependent variable (PD) was assessed using the Kessler Psychological Distress Scale. Job demands were assessed using the Job Content Questionnaire. Working from home was determined by participants’ responses to the question, “Do you currently work from home?” We used a two-level regression analysis adjusted for prefecture. Each individual-level variable at Level 1 was nested into each prefecture at Level 2, stratified by working from home or not. Overall, 21.3% of participants worked from home. The interaction between working from home and job demands was significant. Job demands were positively associated with PD. The stratified analysis indicated that the associations were weaker among employees who worked from home compared with those among employees who did not. The association between job demands and PD may be weakened by working from home.

## 1. Introduction

The ongoing coronavirus disease 2019 (COVID-19) pandemic has accelerated numerous trends regarding the ways in which work is structured, with substantial implications for enterprises, and for worker health and wellbeing [1]. The pandemic poses a threat to psychological health. Previous research revealed profound and wide-ranging psychosocial impacts at the individual, community, and international levels during past outbreaks of infectious diseases [2]. For example, high physical demands and long working hours can result in musculoskeletal pain and disability, whereas unpredictable schedules, employee overload, and occupational stress can contribute to burnout, psychological distress, and cardiovascular problems [3]. The current health crisis has had unprecedented impacts on workplace practices.

High job demands cause depression, which can lead to suicide. Karoshi, or death from overwork, represents a growing public health issue in East Asia [4]. Factors related to COVID-19 infection in the workplace may affect individual coping styles and responses to threats [5]. In addition, previous studies have reported that the number of workplace measures to prevent the spread of infection was positively correlated with worsening respondents’ fears and worries regarding COVID-19 in the workplace [6]. However, workplace-based COVID-19 pandemic countermeasures may weaken the association between job demands and psychological distress.

In response to the COVID-19 pandemic, millions of employees in many countries rapidly shifted to working from home. Several research studies undertaken before the COVID-19 pandemic indicated that telework can reduce work-related stress [7,8,9,10]. In 2020, working from home became crucial for many companies and governments as it allowed people to continue working during the pandemic while reducing the spread of the virus [11]. The positive impacts of working from home, such as reduced commuting time and costs, reduced environmental pollution, and opportunities to support family duties (e.g., picking up children from school), may be desirable for many workers [12]. Employees who worked from home at least 1 day per week were reported to exhibit higher autonomy than those that did not, and to achieve higher levels of flow (enjoyment, absorption, and intrinsic motivation), which was improved by perceived supervisor and collegial support [13]. In addition, the association between telecommuting and psychological distress was found to differ depending on telecommuting preference [14]. 

However, other studies reported increased stress with telework [15,16,17]. During the pandemic situation, control over job situations and predictability were very low. In most cases, workers had no choice regarding the changes to their work as working from home was imposed for safety reasons [18,19,20]. Along with the advantages related to working from home, massive changes in everyday routine and family needs can be a source of intense stress [19]. Protecting and promoting health and well-being when teleworking requires a comprehensive set of measures to provide a healthy and safe work environment, including adequate organization of the work [21,22].

The job demands–resources (JD-R) model conceptualizes job resources as “those physical, psychological, social, or organizational aspects of the job that are either/or: 1. functional in achieving work goals, 2. reduce job demands and the associated physiological and psychological costs, and 3. stimulate personal growth, learning, and development” [23] (p. 2). On the basis of the JD-R model, a previous study showed that working from home affected the association between job demands as well as job resources and well-being [24]. Working from home may therefore be considered as a job resource related to job control.

The third wave of COVID-19 infections in Japan began in December 2020. The current survey was launched on 22 December 2020. By 26 December, there were new record highs for COVID-19 infections, deaths related to the disease, and the number of severe cases, just before the government declared a second state of emergency in the greater Tokyo area on 7 January 2021, almost 1 year since the beginning of the COVID-19 pandemic. This state of emergency was expanded to seven prefectures on 13 January. The third COVID-19 wave may have had different effects on worker mental health than the first and second waves.

**Figure 1:**
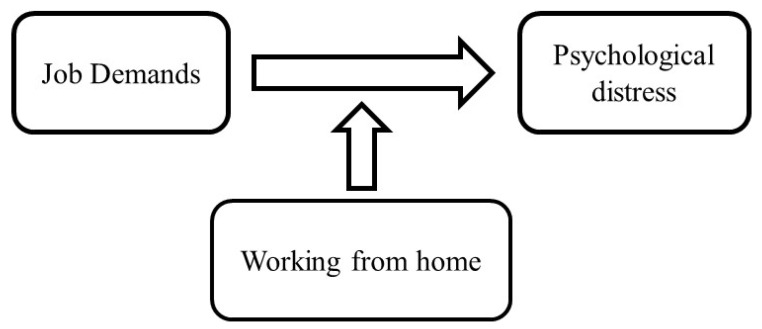
Conceptual model of possible associations between working from home, job demand, and psychological distress.

The present study hypothesized that working from home may weaken the association between high job demands and increased psychological distress among general Japanese workers during the third wave of COVID-19 infections. Figure 1 shows the conceptual model underlying the study hypothesis.

## 2. Materials and Methods

### 2.1. Participants

A cross-sectional online survey was conducted in December 2020 among participants who had previously registered with a Japanese web survey company. Invitations to participate were sent to 665,381 registrants via email. Details of the survey protocol have previously been reported [25]. A sampling plan was designed to recruit an equal number of respondents from 20 collection units comprising a combination of five regions each, with comparable ratios regarding sex and office/non-office worker status. The target sample size was 1500 respondents from each collection unit, giving a total of 30,000 respondents. Thus, 1650 respondents (target sample size plus a margin of 10%) were recruited from each collection unit. Of the 33,302 eligible respondents, 215 were excluded because they were deemed to have provided fraudulent responses by Cross Marketing Inc., leaving 33,087 respondents. The participants were selected using cluster sampling with stratification by sex, region, and job type, and answered the online self-administered questionnaire. Participants were selected using a random number generator. The study population comprised individuals interested in participating in a survey. There was a modest financial incentive for survey participation (equivalent to a few US dollars). We excluded 6051 surveys with invalid responses or response errors, leaving 27,036 surveys for analysis in this study. The exclusion criteria were: extremely short response time (≤6 min), extremely low body weight (<30 kg), extremely short height (<140 cm), inconsistent answers to similar questions throughout the survey (e.g., inconsistent responses to questions about marital status and living area), and incorrect answers to a staged question used to identify fraudulent responses (i.e., “Choose the third-largest number from the following five numbers”). 

The study aims and protocol were approved by the Ethics Committee of Medical Research, University of Occupational and Environmental Health, Japan (R2-079). Informed consent to participate in this study was obtained from all participants. Participants were informed in advance that their participation was strictly voluntary and all information they provided would remain confidential. Individuals who consented to participate were able to access a designated website (after confirmation of their personal information) where they could complete the survey. Participants had the option to not respond to any part of the questionnaire and could discontinue participation at any time.

### 2.2. Measures

#### 2.2.1. Dependent Variable: Psychological Distress

Psychological distress was assessed using the Kessler Psychological Distress Scale (K6). The K6 was originally developed as a screening instrument for non-specific psychological distress and serious mental illness. Its internal reliability and validity have been documented [26]. The K6 comprises a six-item battery asking how frequently respondents had experienced specific symptoms of psychological distress in the past 30 days such as “During the last 30 days, about how often did you feel nervous?” and “During the last 30 days, about how often did you feel hopeless?” Possible responses ranged from 0 (none of the time) to 4 (all of the time), giving a total score of 0–24. The K6 has been translated into Japanese, and the Japanese version has been validated [26]. In this sample, the Cronbach’s α coefficient for the K6 was 0.88.

#### 2.2.2. Independent Variable: Job Demands

We used the job demands scale from the Japanese version of the Job Content Questionnaire (JCQ) [27]. The JCQ was developed by Karasek and is based on the job demands–control (or demand–control–support) model. It contains five items, including “My job requires working very fast” and “My job requires working very hard” (response range: 12–48), that assess job demands, rated on a four-point scale (1 = strongly disagree to 4 = strongly agree). The total score was calculated according to the JCQ User’s Guide (score range: 12–48) [28]. The Japanese version of the JCQ had acceptable reliability and validity [27]. In the present study, the Cronbach’s α coefficient for job demands was 0.68.

#### 2.2.3. Moderator Variable: Working from Home

Working from home was determined by participants’ responses to the question, “Do you currently work from home?” Response options were “More than 4 days per week”, “More than 2 days per week”, “Less than 1 day per week”, and “Hardly ever”. Responses were subsequently dichotomized using a two-point scale: 0 = yes (“More than 4 days per week”, “From more than 2 days per week to less than 3 days per week”, “Less than 1 day per week”); and 1 = no (“Hardly ever”).

#### 2.2.4. Assessment of Covariates

Covariates were measured using a self-administered questionnaire and included demographic and lifestyle characteristics such as sex, age, marital status, educational attainment, occupation, job type, annual family income, and company size. Age was expressed as a continuous variable. Marital status was classified into three categories: married, divorced/widowed, and unmarried. Educational attainment was classified into three categories: junior high school and high school, college and technical school, and university and graduate school. Occupation was classified into 10 categories: staff member; manager; executive; public official/teaching staff/non-profit organization employee; temporary and contract employee; self-employed person; small office/home office worker; agriculture, forestry, and fishery worker; professional (e.g., lawyer, accountant, medical doctor); and others. Job type was classified into three categories: mainly desk work (clerical or computer work), mainly talking to people (e.g., customer service, sales, selling), and mainly labor (e.g., work at construction sites, physical work, nursing care). Participants were asked to indicate their yearly equivalent household income by choosing one of five income bands: (i) 47.4–224.5 million JPY; (ii) 225.0–317.5 million JPY; (iii) 318.1–428.7 million JPY; (iv) 433.0–525.0 million JPY; and (v) 530.3–1050.0 million JPY. Company size was categorized into 10 groups by number of employees: 1 (self-employment), 2–4, 5–9, 10–29, 30–49, 50–99, 100–499, 500–999, 1000–9999, and ≥10,000 employees. The cumulative incidence rate of COVID-19 infection 1 week before the survey in the residential prefectures was used as a prefecture-level variable. This information was collected from the websites of public institutions.

### 2.3. Statistical Analyses

Student’s *t*-tests and chi-square tests were used to examine differences in demographic variables and psychological distress between participants who were working from home and those who were not. We used multilevel regression analyses with two levels adjusted for the prefectural level, whereby each individual-level variable at level 1 was nested into each prefecture at level 2. Examination of the interaction between working from home and job demands revealed a significant interaction (*p* = 0.02). To compare the adjusted coefficients by presence or absence of working from home, multiple regression analyses were used to examine the association between job demands and psychological distress stratified by availability of telecommuting. We conducted multiple regression analysis using a crude model (Model 1) and a model adjusted for sex, age, marital status, educational attainment, occupation, job type, annual household income, and company size (Model 2). In addition, to compare the adjusted coefficients of the interaction between the availability of telecommuting and job demand by sex and age, multiple regression analyses were used to examine the interaction between the availability of telecommuting and job demand stratified by sex and age (divided by two groups: “less than 40” and “40 or more”). All analyses were performed using Stata 15SE (StataCorp, College Station, TX, USA), with statistical significance set at *p* < 0.05.

## 3. Results

Approximately 20% of participants had the opportunity to work from home. Employees who worked from home were older and had lower psychological distress than those who did not work from home. Men, self-employed people, those with a higher household income, those whose job mainly involved desk work, and employees in large companies were more likely to work from home (Table 1). 

Multiple regression analysis for all participants with job demands, working from home, and other variables as independent variables showed that both job demands and working from home had significant independent effects on psychological distress (coefficient = 0.17, *p* < 0.01 and coefficient = 0.24, *p* < 0.01, respectively). The stratified analysis (post-hoc simple slope analysis) showed that the effect of job demands on psychological distress was weaker among employees who worked from home (coefficient = 0.15) than among those who did not (coefficient = 0.18) (Table 2). Company size was positively associated with psychological distress among those who worked from home, and negatively associated with psychological distress among those who did not work from home.

We confirmed an interaction between job demands, working from home, and sex (*p* < 0.05), and an interaction between job demands, working from home, and age (*p* < 0.05). Regarding sex, the stratified analysis showed that the effect of interactions between job demand and working from home was significant among men (coefficient −0.07, *p* < 0.05) but was not significant among women (coefficient 0.001, *p* = 0.96). Regarding age, the stratified analysis showed that the effect of interactions between job demand and working from home was significant among employees aged 40 and over (coefficient −0.05, *p* < 0.05) but was not significant among employees under 40 years old (coefficient 0.04, *p* = 0.21).

## 4. Discussion

We conducted a large online survey on 22 December 2020, just before the government declared a second state of emergency in the greater Tokyo area (7 January 2021). Men, self-employed people, those with a higher household income, those whose job mainly involved desk work, and employees in large companies were more likely to work from home. The association between job demands and psychological distress was stronger among employees who did not work from home compared with that among employees who worked from home.

Working from home was found to reduce the psychological distress, and the association between job demand and psychological distress was weakened by working from home. Previous research has suggested that working from home may weaken the association between job demands and psychological distress. In one study, working from home was associated with reduced commuting time and costs, reduced environmental pollution, and the opportunity to support family duties (e.g., picking up children from school), which may be desirable for many workers [12]. Having a sense of control over worktime can also help employees manage their work–life balance [29]. Irrespective of the COVID-19 pandemic, many employers can proactively provide opportunities to work from home to prevent psychological distress among employees. In addition, working from home may reduce the fear of infection at or on the way to work [30,31]. Our previous study showed that the association between job demands and psychological distress was strengthened by anxiety about COVID-19 infection in the workplace [5]. Working from home may affect the association between job demands and psychological distress through reducing anxiety about COVID-19 infection.

The effect of company size on psychological distress differed between those who worked from home and those that did not. The association between company size and psychological distress in previous studies was inconsistent [32,33]. This discrepancy may be attributable to the use of different indicators (company size vs. worksite size) or different survey years reflecting different economic situations. Working from home decreased employees’ communication with their supervisors and colleagues [34]. Therefore, working from home may affect the association between company size and psychological distress via communication changes.

The interactional effects between job demands and working from home differed by sex and age. Women and younger employees had worse adjustment outcomes during working from home [19]. In addition, women reported higher perceived interference of work on family needs. The differences in psychological impacts may reflect traditional gender roles [35]. In younger employees, excessive engagement in working activities can account for a higher achievement orientation and desire for self-esteem [36]. Indeed, forced sudden working from home organization has abruptly pushed employees to rearrange behaviors, habits, and communication styles [21]. Several structural, logistic, and technological inconveniences may have caused a longer time to work and greater involvement in job-related tasks [19]. For women and younger employees, working from home may almost eliminate the boundary between work and private life/home, which may make it difficult to maintain the balance between them.

The current study involved several limitations. First, our study population required Internet access to complete the survey and therefore might have comprised participants that were more aware of COVID-19 infection through access to online information. People should be aware of the psychological risks of excessive media exposure and control their access in health crises such as the COVID-19 outbreak [37]. Our results are not completely generalizable to individuals without Internet access or to people in other countries and settings. Second, we had no information about the participants’ personality traits. In addition, we had no information about the number of confirmed cases of COVID-19 in the respondents’ workplaces, or information about the respondents’ previous experience working from home before the COVID-19 pandemic. Further studies are needed to evaluate whether other confounding factors provide possible mechanisms for the observed attenuation in the associations between job demands, working from home, and psychological distress. Third, this study used a cross-sectional design, and no causal associations could be determined. A further study using an interventional or prospective design is needed to clarify the potential causal associations between job demands, working from home, and psychological distress in the Japanese working population. Finally, we should consider the possible effects of common method bias when interpreting the results.

## 5. Conclusions

Working from home was found to reduce the psychological distress, and the association between job demand and psychological distress may be weakened by working from home. The effect of company size on psychological distress differed between those who worked from home and those that did not. The interactional effects between job demand and working from home differed by sex and age. The effect of working from home on employees’ health remains unclear because of the large number of factors related to the association.

## Figures and Tables

**Table 1:** Participants’ characteristics (N = 27,036).

	Working from Home				
	Yes (n = 5760)		No (n = 21,276)		*p*
Age, years (SD)	48.9	(10.2)	46.5	(10.6)	<0.001 ^a^
Range (minimum–maximum)	20–65		20–65		
Job demand, Job Content Questionnaire score (SD)	29.1	(6.0)	30.3	(5.6)	<0.001 ^a^
Range (minimum–maximum)	12–48		12–48		
Psychological distress, K6 score (SD)	4.5	(5.7)	4.7	(5.0)	0.04
Range (minimum–maximum)	0–24		0–24		
Sex					
Men	3361	(58.4)	10,453	(49.1)	<0.001 ^b^
Women	2399	(41.7)	10,823	(50.9)	
Marital status					
Married	3280	(56.9)	11,479	(55.2)	0.014 ^b^
Divorced or widowed	504	(8.8)	2339	(11.0)	
Unmarried	1976	(34.3)	7188	(33.8)	
Educational attainment, n (%)					
Junior high school and high school	993	(17.2)	6328	(29.7)	<0.001 ^b^
College and technical school	1132	(19.7)	5412	(25.4)	
University and graduate school	3635	(63.1)	9536	(44.8)	
Occupation, n (%)					
Staff member	2082	(36.2)	10,493	(49.3)	<0.001 ^b^
Manager	796	(13.8)	1745	(8.2)	
Executive	273	(4.7)	589	(2.8)	
Public official, teaching staff, and non-profit organization employees	248	(4.3)	2562	(12.0)	
Temporary and contract employees	416	(7.2)	2478	(11.7)	
Self-employed person	1055	(18.3)	1174	(5.5)	
Small office home office worker	330	(5.7)	48	(0.2)	
Agriculture, forestry, and fisheries	54	(0.9)	158	(0.7)	
Professionals (e.g., lawyer, accountant, medical doctor)	153	(2.7)	1694	(8.0)	
Other	353	(6.1)	335	(1.6)	
Job type					
Mainly desk work (clerical or computer work)	4052	(70.4)	9416	(44.3)	<0.001 ^b^
Mainly talking to people (customer service, sales, selling)	1219	(21.2)	5708	(26.8)	
Mainly labor (work at production sites, physical work, nursing care)	489	(8.5)	6152	(28.9)	
Yearly household income, million JPY, n (%)					
1 (47.4–224.5)	1049	(18.2)	4054	(19.1)	<0.001 ^b^
2 (225.0–317.5)	922	(16.0)	4690	(22.0)	
3 (318.1–428.7)	944	(16.4)	4317	(20.3)	
4 (433.0––525.0)	1119	(19.4)	3914	(18.4)	
5 (530.3–1050.0)	1726	(30.0)	4301	(20.2)	
Company size, employees, n (%)					
1 (self-employment)	1518	(26.4)	1038	(4.7)	<0.001 ^b^
2–4	557	(9.7)	1439	(6.8)	
5–9	213	(3.7)	1400	(6.6)	
10–29	270	(3.7)	2528	(11.9)	
30–49	160	(4.7)	1432	(6.7)	
50–99	273	(2.8)	2277	(10.7)	
100–499	668	(11.6)	4488	(21.1)	
500–999	363	(6.3)	1634	(7.7)	
1000–9999	1076	(18.7)	3643	(17.1)	
≥10,000	662	(11.5)	1397	(6.6)	

^a^ Student’s *t*-test, ^b^ chi square test; SD, standard deviation; K6, Kessler Psychological Distress Scale.

**Table 2:** Associations between job demand and psychological distress by working from home (N = 27,036).

Working from Home	Yes (n = 5760)				No (n = 21,276)			
	Model 1		Model 2		Model 1		Model 2	
	Coefficient	95%CI	Coefficient	95%CI	Coefficient	95%CI	Coefficient	95%CI
Job demand	0.15	(0.13–0.17)	0.15	(0.13–0.18)	0.19	(0.17–0.20)	0.18	(0.17–0.19)
Sex	1.16	(0.88–1.44)	0.28	(−0.04–0.59)	1.16	(1.01–1.31)	0.13	(−0.04–0.31)
Age	−0.10	(−0.11–−0.09)	−0.07	(−0.09–−0.06)	−0.09	(−0.10–−0.08)	−0.06	(−0.07–−0.05)
Marriage								
Married	ref		Ref		ref		ref	
Divorced or widowed	0.81	(0.31–1.31)	0.75	(0.26–1.24)	1.18	(0.94–1.42)	0.88	(0.64–1.12)
Unmarried	1.66	(1.37–1.96)	0.92	(0.61–1.24)	1.69	(1.53–1.85)	0.99	(0.82–1.15)
Educational attainment								
Junior high school and high school	ref		Ref		ref		ref	
College and technical school	0.14	(−0.32–0.59)	−0.06	(−0.50–0.38)	0.23	(0.03–0.43)	0.03	(−0.17–0.23)
University and graduate school	−0.19	(−0.56–0.19)	0.01	(−0.37–0.38)	−0.27	(−0.45–−0.10)	−0.11	(−0.29–−0.07)
Occupation								
Staff member	ref		Ref		ref		ref	
Manager	−1.05	(−1.48–−0.61)	0.02	(−0.42–0.47)	−1.30	(−1.57–−1.03)	−0.30	(−0.58–−0.02)
Executive	−1.87	(−2.54–−1.19)	−0.96	(−1.67–−0.25)	−1.92	(−2.37–−1.47)	−0.47	(−0.93–−0.02)
Public officials, teaching staff, and non-profit organization employees	0.28	(−0.43–0.98)	0.74	(0.06–1.42)	−0.67	(−0.91–−0.44)	−0.18	(−0.42–0.06)
Temporary and contract employees	−0.14	(−0.70–0.42)	−0.01	(−0.56–0.55)	0.18	(−0.06–0.42)	0.30	(0.06–0.54)
Self-employed person	−0.46	(−0.86–−0.06)	−0.49	(−1.05–0.07)	−1.22	(−1.55–−0.90)	−0.26	(−0.61–0.10)
Small Office Home Office worker	0.33	(−0.29–0.95)	−0.19	(−0.92–0.54)	−1.41	(−2.95–0.12)	−0.72	(−2.21–0.77)
Agriculture, forestry and fisheries	0.12	(−1.33–1.56)	−0.37	(−1.87–1.12)	−0.49	(−1.34–0.36)	0.09	(−0.75–0.93)
Professionals (Lawler, accountant, medical doctor, etc.)	−0.27	(−1.15–0.61)	−0.36	(−1.28–0.55)	−0.31	(−0.59–−0.03)	−0.58	(−0.87–−0.30)
Other	0.57	(−0.03–1.18)	−0.16	(−0.86–0.55)	−0.44	(−1.03–0.15)	−0.19	(−0.77–0.39)
Job type								
Mainly desk work (clerical or computer work)	ref		Ref		ref		ref	
Mainly talking to people (customer service, sales, selling, etc.)	−0.11	(−0.45–0.24)	−0.22	(−0.56–0.12)	0.12	(−0.06–0.30)	−0.36	(−0.54–−0.18)
Mainly labor (work at production sites, physical work, nursing care, etc.)	0.46	(−0.05–0.96)	−0.08	(−0.60–0.44)	0.32	(0.14–0.49)	−0.22	(−0.41–−0.04)
Yearly household income	−0.49	(−0.58–−0.40)	−0.42	(−0.52–−0.32)	−0.43	(−0.48–−0.38)	−0.32	(−0.38–−0.27)
Company size	−0.06	(−0.10–−0.01)	−0.11	(−0.18–−0.04)	0.07	(0.05–0.10)	0.03	(0.00–0.06)

Model 1: Crude model. Model 2: Adjusted for sex, age, marriage, educational attainment, occupation, job type, annual household income, and company size. CI, confidence interval.

## Data Availability

The data presented in this study are not available to the public due to ethical restrictions.

## References

[1] Peters S.E., Dennerlein J.T., Wagner G.R., Sorensen G. (2022). Work and worker health in the post-pandemic world: A public health perspective. Lancet Public Health.

[2] Mukhtar S. (2020). Psychological health during the coronavirus disease 2019 pandemic outbreak. Int. J. Soc. Psychiatry.

[3] Kelly E.L., Moen P. (2021). Review of Overload: How Good Jobs Went Bad and What We Can Do about It.

[4] Eguchi H., Wada K., Smith D.R. (2016). Recognition, compensation, and prevention of karoshi, or death due to overwork. J. Occup. Environ. Med..

[5] Eguchi H., Hino A., Inoue A., Tsuji M., Tateishi S., Ando H., Nagata T., Matsuda S., Fujino Y. (2021). Effect of anxiety about COVID-19 infection in the workplace on the association between job demands and psychological distress. Front. Public Health.

[6] Sasaki N., Kuroda R., Tsuno K., Kawakami N. (2020). Workplace responses to COVID-19 associated with mental health and work performance of employees in Japan. J. Occup. Health.

[7] Allen T.D., Golden T.D., Shockley K.M. (2015). How effective is telecommuting? Assessing the status of our scientific findings. Psychol. Sci. Public Interest.

[8] Arvola R., Tint P., Kristjuhan Ü., Siirak V. (2017). Impact of telework on the perceived work environment of older workers. Sci. Ann. Econ. Bus..

[9] Bosua R., Gloet M., Kurnia S., Mendoza A., Yong J. Telework, Productivity and Wellbeing: An Australian Perspective. Institute for a Broadband-Enabled Society 2012. https://research.unimelb.edu.au/__data/assets/pdf_file/0029/147818/IBES-2013-Annual-Report.pdf.

[10] Gajendran R.S., Harrison D.A. (2007). The good, the bad, and the unknown about telecommuting: Meta-analysis of psychological mediators and individual consequences. J. Appl. Psychol..

[11] Şentürk E., Sağaltıcı E., Geniş B., Günday Toker Ö. (2021). Predictors of depression, anxiety and stress among remote workers during the COVID-19 pandemic. Work.

[12] Kotera Y., Correa Vione K. (2020). Psychological impacts of the new ways of working (NWW): A systematic review. Int. J. Environ. Res. Public Health.

[13] Peters P., Poutsma E., Van der Heijden B.I.J.M., Bakker A.B., Bruijn T.D. (2014). Enjoying new ways to work: An HRM-process approach to study flow. Hum. Resour. Manag..

[14] Otsuka S., Ishimaru T., Nagata M., Tateishi S., Eguchi H., Tsuji M., Ogami A., Matsuda S., Fujino Y. (2021). A Cross-sectional study of the mismatch between telecommuting preference and frequency associated with psychological distress among Japanese workers in the COVID-19 pandemic. J. Occup. Environ. Med..

[15] de Macêdo T.A.M., Cabral E., Silva Castro W.R., de Souza Junior C.C., da Costa Junior J.F., Pedrosa F.M., da Silva A.B., de Medeiros V.R.F., de Souza R.P., Cabral M.A.L. (2020). Ergonomics and telework: A systematic review. Work.

[16] Heiden M., Widar L., Wiitavaara B., Boman E. (2021). Telework in academia: Associations with health and well-being among staff. High. Educ..

[17] Song Y., Gao J. (2020). Does telework stress employees out? A study on working at home and subjective well-being for wage/salary workers. J. Happiness Stud..

[18] Spagnoli P., Molinaro D. (2020). Negative (workaholic) emotions and emotional exhaustion: Might job autonomy have played a strategic role in workers with responsibility during the COVID-19 crisis lockdown?. Behav. Sci..

[19] Orfei M.D., Porcari D.E., D’Arcangelo S., Maggi F., Russignaga D., Lattanzi N., Malizia A.P., Ricciardi E. (2022). COVID-19 and stressful adjustment to work: A long-term prospective study about homeworking for bank employees in Italy. Front. Psychol..

[20] Orfei M.D., Bossi F., D’Arcangelo S., Maggi F., Lattanzi N., Malizia A.P., Ricciardi E. (2022). Mental health in the post-lockdown pandemic phase: Relief or exacerbation of psychological distress? A cross-sectional study in the general population in Italy. Acta Psychol..

[21] World Health Organization and International Labour Organization (2022). Health and Safe Telework: Technical Brief. https://www.who.int/publications/i/item/9789240040977.

[22] Beckel J.L.O., Fisher G.G. (2022). Telework and worker health and well-being: A review and recommendations for research and practice. Int. J. Environ. Res. Public Health.

[23] Demerouti E., Bakker A.B. (2011). The job demands-resources model: Challenges for future research. SA J. Ind. Psychol..

[24] Da S., Fladmark S.F., Wara I., Christensen M., Innstrand S.T. (2022). To change or not to change: A study of workplace change during the COVID-19 pandemic. Int. J. Environ. Res. Public Health.

[25] Fujino Y., Ishimaru T., Eguchi H., Tsuji M., Tateishi S., Ogami A., Mori K., Matsuda S. (2021). Protocol for a nationwide internet-based health survey of workers during the COVID-19 pandemic in 2020. J. UOEH.

[26] Furukawa T.A., Kawakami N., Saitoh M., Ono Y., Nakane Y., Nakamura Y., Tachimori H., Iwata N., Uda H., Nakane H. (2008). The performance of the Japanese version of the K6 and K10 in the World Mental Health Survey Japan. Int. J. Methods Psychiatr. Res..

[27] Kawakami N., Kobayashi F., Araki S., Haratani T., Furui H. (1995). Assessment of job stress dimensions based on the job demands-control model of employees of telecommunication and electric power companies in Japan: Reliability and validity of the Japanese version of the Job Content Questionnaire. Int. J. Behav. Med..

[28] Karasek R., Brisson C., Kawakami N., Houtman I., Bongers P., Amick B. (1998). The Job Content Questionnaire (JCQ): An instrument for internationally comparative assessments of psychosocial job characteristics. J. Occup. Health Psychol..

[29] Beckers D.G., Kompier M.A., Kecklund G., Härmä M. (2012). Worktime control: Theoretical conceptualization, current empirical knowledge, and research agenda. Scand. J. Work Environ. Health.

[30] Nguyen M.H. (2021). Factors influencing home-based telework in Hanoi (Vietnam) during and after the COVID-19 era. Transportation.

[31] Sato K., Sakata R., Murayama C., Yamaguchi M., Matsuoka Y., Kondo N. (2021). Changes in work and life patterns associated with depressive symptoms during the COVID-19 pandemic: An observational study of health app (CALO mama) users. Occup Environ. Med..

[32] Inoue A., Kawakami N., Tsuchiya M., Sakurai K., Hashimoto H. (2010). Association of occupation, employment contract, and company size with mental health in a national representative sample of employees in Japan. J. Occup. Health.

[33] Kanamori S., Tsuji T., Takamiya T., Kikuchi H., Inoue S., Takagi D., Kai Y., Yamakita M., Kameda Y., Kondo K. (2020). Size of company of the longest-held job and mortality in older Japanese adults: A 6-year follow-up study from the Japan Gerontological Evaluation Study. J. Occup. Health.

[34] Amano H., Fukuda Y., Shibuya K., Ozaki A., Tabuchi T. (2021). Factors associated with the work engagement of employees working from home during the COVID-19 pandemic in Japan. Int. J. Environ. Res. Public Health.

[35] Oakman J., Kinsman N., Stuckey R., Graham M., Weale V. (2020). A rapid review of mental and physical health effects of working at home: How do we optimise health?. BMC Public Health.

[36] Ng T.W.H., Sorensen K.L., Feldman D.C. (2007). Dimensions, antecedents, and consequences of workaholism: A conceptual integration and extension. J. Organ. Behav..

[37] Sasaki N., Kuroda R., Tsuno K., Kawakami N. (2020). Exposure to media and fear and worry about COVID-19. Psychiatry Clin. Neurosci..

